# Multifaceted surgical approach of combined thoracoretroperitoneal incision and midline abdominal incision for a secondary aortoenteric fistula

**DOI:** 10.1186/s13019-024-02496-2

**Published:** 2024-01-28

**Authors:** Tomoyuki Minami, Takahiro Kojima, Naoto Yabu, Ichiya Yamazaki, Aya Saito

**Affiliations:** 1 Cardiovascular Surgery, Fujisawa City Hospital, Fujisawa 2-6-1, Fujisawa, 251-8550 Kanagawa Japan; 2 Department of Surgery, Yokohama City University, Fukuura 3-9, Kanazawa-ku, Yokohama, 236-004 Kanagawa Japan

**Keywords:** Secondary aortoenteric fistula, Thoracoretroperitoneal incision, Midline abdominal incision

## Abstract

**Background:**

We report a one-stage surgery to the case of secondary aortoenteric fistula (sAEF) after prosthetic reconstruction of abdominal aortic aneurysm, by multifaceted approach.

**Case presentation:**

A 63-year-old male was admitted to our unit under diagnosed of sAEF after prosthetic reconstruction of abdominal aortic aneurysm, and a pseudoaneurysm of thoracoabdominal aorta due to infection. The patient underwent emergency operation. Firstly, we placed the patient in a modified right lateral decubitus position and performed thoracoabdominal aortic replacement with retroperitoneal approach by thoracoretroperitoneal incision which combined thoracotomy and pararectal incision, and secondly, we changed to a supine position and performed closure of the duodenal fistula and omental flap transposition by midline abdominal incision. The patient was doing well without complications.

**Conclusions:**

A one-stage, multifaceted surgical approach covering both prosthetic reconstruction of thoracoabdominal aorta and closure of sAEF with omentopexy is reasonable and useful strategy.

## Background

Secondary aortoenteric fistula (sAEF) after prosthetic reconstruction of abdominal aortic aneurysm is a relatively rare complication; however, it is difficult to diagnose and is fatal [1, 2]. Massive bleeding from an aortoenteric fistula and sepsis due to prosthetic graft infection lead to a serious morbidity and high mortality; thus, early treatment is required. Surgery is the first treatment of choice, but treatment for both the aorta and intestinal tract is required, and an optimal operative method has not been established.

In this case report, we report a one-stage surgery to the case of sAEF after prosthetic reconstruction of abdominal aortic aneurysm by multifaceted approach combined thoracoretroperitoneal incision and midline abdominal incision. We safely replaced the thoracoabdominal aorta and safely closed the duodenal fistula.

## Case presentation

**Fig. 1:**
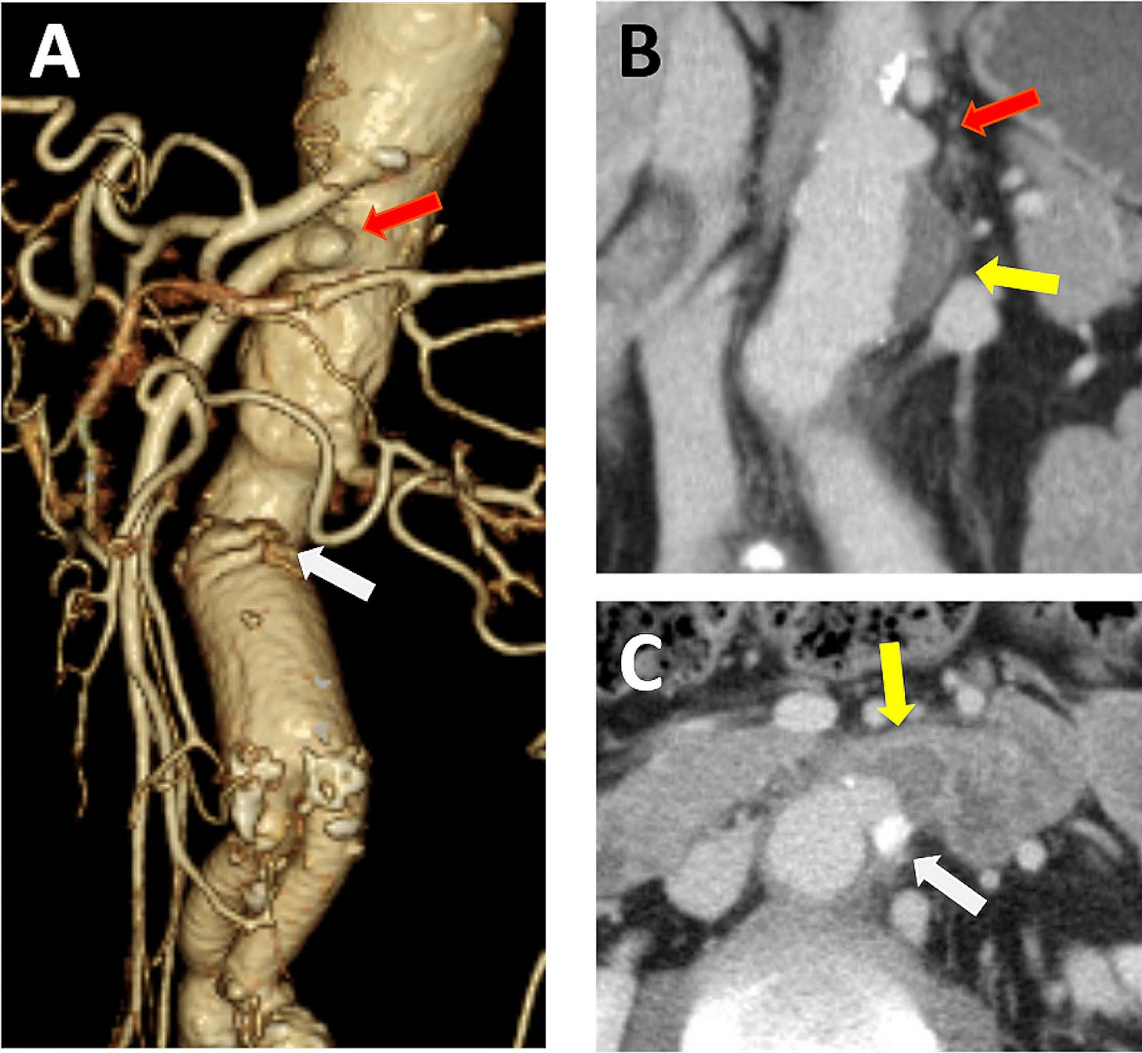
Preoperative computed tomography. **A** Three-dimensional view showing a pseudoaneurysm of the anastomotic site (white arrow) and a pseudoaneurysm at the superior mesenteric artery bifurcation (red arrow). **B** Coronal view demonstrating a pseudoaneurysm at the superior mesenteric artery bifurcation (red arrow) and atheroma on the central side of the anastomotic site (yellow arrow). **C** Axial view demonstrating a pseudoaneurysm of the anastomotic site (white arrow) and soft tissue that connected the horizontal part of the duodenum (yellow arrow)

A 63-year-old male with hypertension complained of hematemesis and hematochezia and sought consult at our emergency department. He had undergone prosthetic reconstruction for infrarenal abdominal aortic aneurysm by midline abdominal incision at another hospital 8 years prior. He had no fever and no evidence of elevated inflammatory response. His blood pressure was 80/50mmHg and he was in shock. Emergency upper gastrointestinal endoscopy was performed; however, the bleeding source could not be identified. Contrast-enhanced computed tomography (CT) showed soft tissue that connected the horizontal part of the duodenum at the anastomotic site of the artificial blood vessel, and a pseudoaneurysm of the anastomotic site was suspected. Furthermore, a pseudoaneurysm was found at the superior mesenteric artery bifurcation on the central side of the anastomotic site, suggesting a complication of infection (Fig. 1). A secondary aortoenteric fistula (sAEF) was diagnosed after prosthetic reconstruction of the abdominal aortic aneurysm and a pseudoaneurysm of thoracoabdominal aorta. Emergency operation was performed. Firstly, we performed thoracoabdominal aortic replacement by thoracoretroperitoneal incision, and secondly, we changed his body position and performed closure of the duodenal fistula and omental flap transposition by midline abdominal incision.

**Fig. 2:**
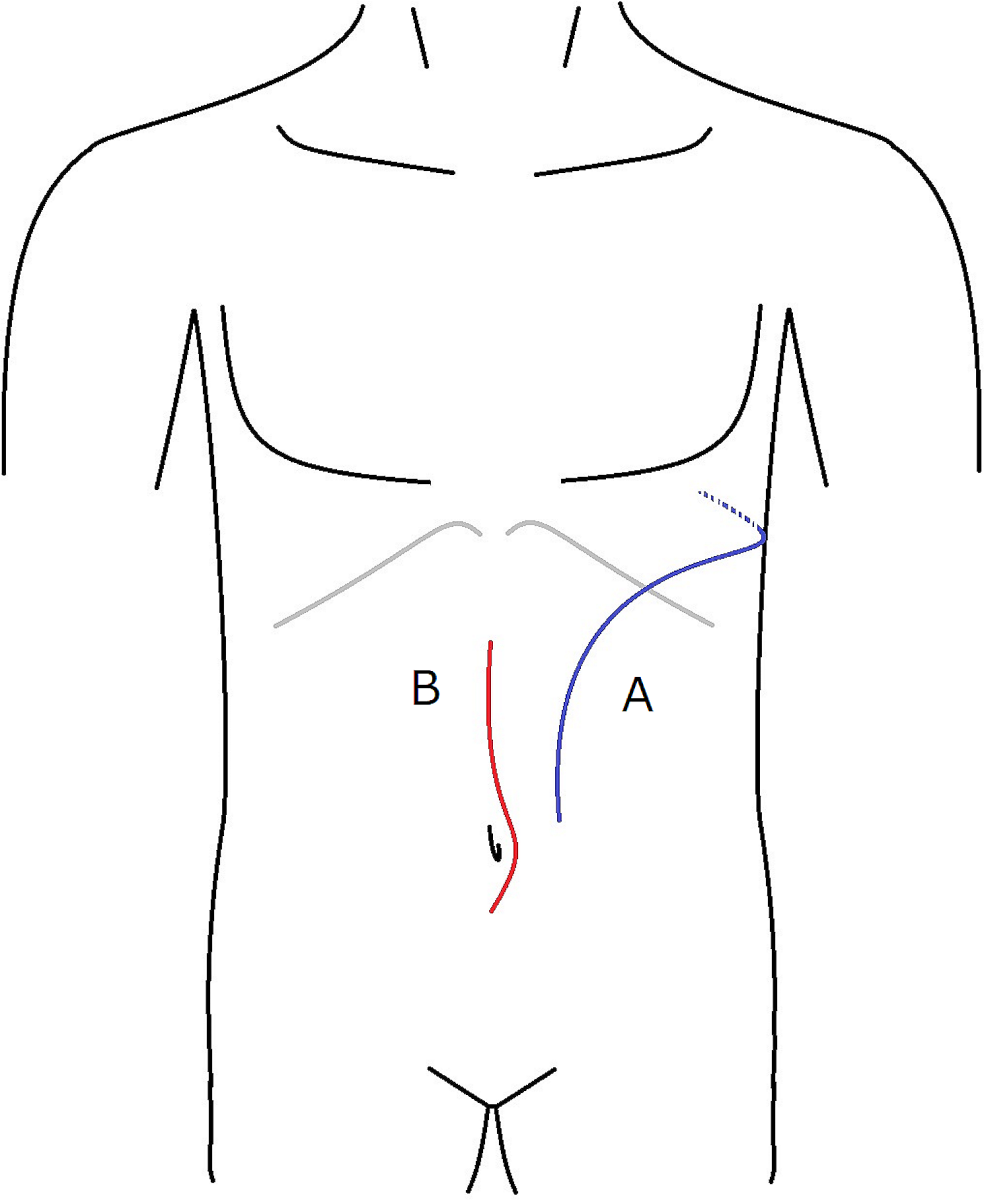
Incisions on a supine patient. **A** (blue line) Thoracoretroperitoneal incision which combined a left seventh intercostal thoracotomy and pararectal incision. **B** (red line) Median upper-middle incision

The patient was placed in a modified right lateral decubitus position, with the left shoulder at 50 to 70 degrees to the operating table, and the pelvis rotated posteriorly as fa as possible (corkscrew position) and a left inguinal incision was made to secure the femoral artery and vein. We performed thoracoretroperitoneal incision which combined a left seventh intercostal thoracotomy and pararectal incision (Fig. 2A). The aorta was reached via a retroperitoneal approach. Firstly, the descending aorta was secured. Then, the retroperitoneum was dissected and the intra-abdominal organs were displaced with the peritoneum. The Y-shaped artificial blood vessel that had been replaced was confirmed and was tightly adherent. The straight portion of the artificial blood vessel was secured. We dissected the proximal aorta and secured the left renal artery, celiac artery, and superior mesenteric artery.

After heparin was administered, veno-arterial extracorporeal membrane oxygenation (V-A ECMO) was started using the left femoral artery and vein. The descending aorta and straight portion of the artificial blood vessel were clamped and the dorsal side of the aorta was incised. 12 French balloon-tipped catheters extending from the V-A ECMO were inserted into the celiac artery, superior mesenteric artery, and renal arteries for selective visceral perfusion. After the aortic wall was resected and cleaned, we used a Gelweave Coselli thoracoabdominal graft (Vascutek-Terumo, Renfrewshire Scotland, UK) bonded with rifampicin and the proximal and distal anastomosis were performed before the visceral arteries are reattached. The Distal anastomosis was performed at the straight portion of the artificial blood vessel where there were no obvious signs of infection. After the clamp of the aorta was released, the renal, the celiac, the superior mesenteric anastomoses were carried out. After thoracoabdominal aortic replacement was performed, V-A ECMO was discontinued and protamine was administered and hemostasis was performed.

**Fig. 3:**
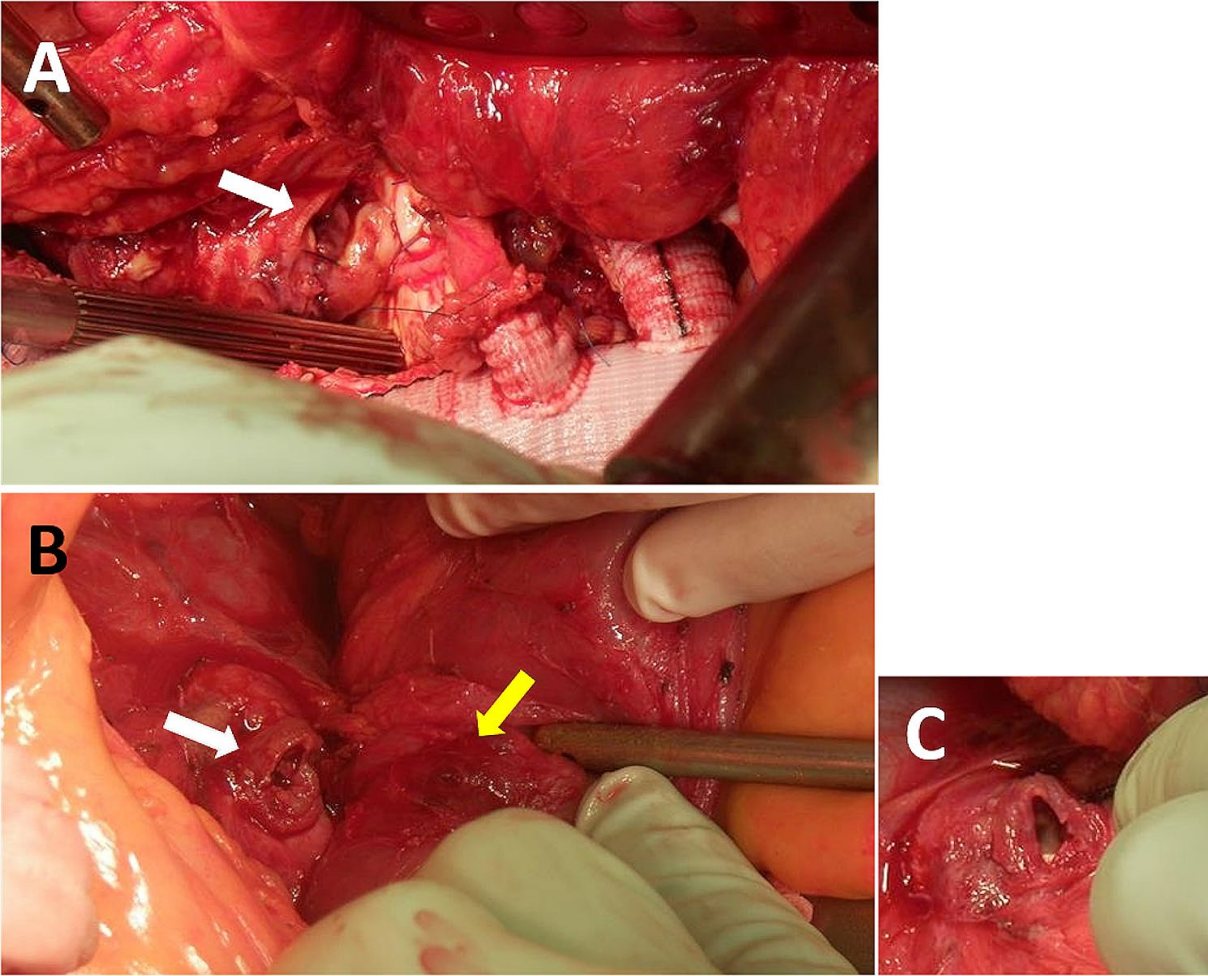
Intraoperative photo. **A** In a right lateral decubitus position, a left seventh intercostal thoracotomy and a retroperitoneal approach were performed. Thoracoabdominal aortic replacement was performed using a graft bonded with rifampicin.　 The lumen of the anastomosis of the pseudoaneurysm was confirmed (white arrow). **B** In a supine position and midline abdominal incision, the retroperitoneal perforation (white arrow) and an 8-mm pinhole fistula in the duodenum (yellow arrow) were confirmed. **C** The new artificial vessel was found inside the retroperitoneal perforation

We considered complete artificial blood vessel resection around the aortoenteric fistula; however, a part of the artificial blood vessel around the aortoenteric fistula tightly adhered to the retroperitoneum and we were concerned about intestinal perforation; thus, a part of the artificial blood vessel was left in place (Fig. 3A).

After rinsing with saline, a drain was placed and the chest and the thoracoretroperitoneal incision were closed.

The patient was then placed in a supine position and the abdomen was opened through a median upper-middle incision (Fig. 2B). The duodenum was rigidly attached to the retroperitoneum at the position of the aorta. This was considered as the fistula site. The fistula was detached closer to the retroperitoneum, and the duodenum was detached from the artificial blood vessel (Fig. 3B). The previous artificial blood vessel was trimmed and completely resected and the new artificial vessel was found inside the retroperitoneal perforation (Fig. 3C). The duodenal fistula site only had an 8-mm serosal membrane injury and a pinhole in the center (Fig. 3B). The fistula was closed using two layers of direct sutures because the condition of the duodenal wall was good. The space between the new artificial blood vessel and the duodenum was filled with the omental flap and the omental flap was filled as much as possible around the graft through the retroperitoneal perforator. After repair of the duodenum, an enterostomy was created.

After rinsing with saline, a drain was inserted and the median upper-middle incision was closed.

**Fig. 4:**
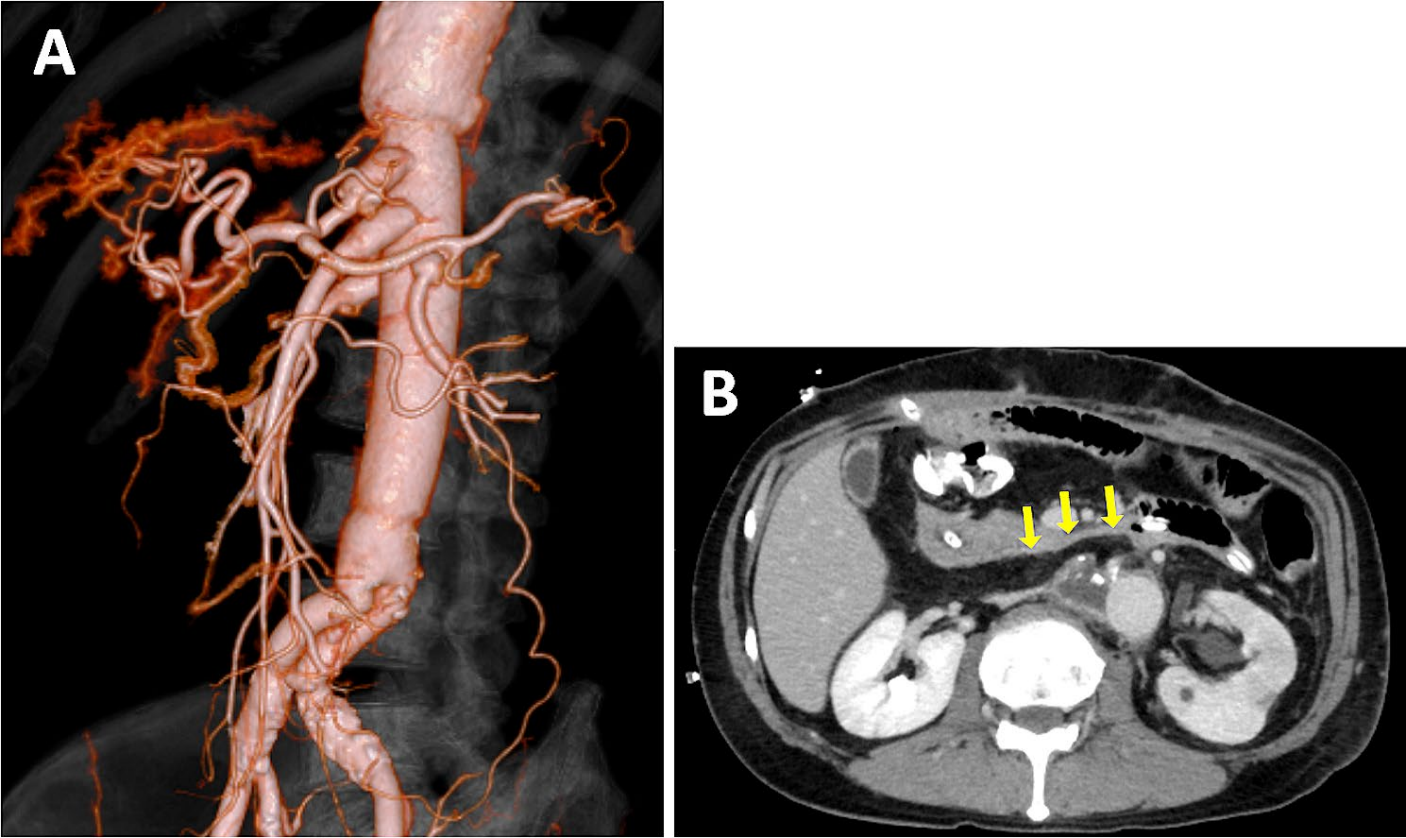
Postoperative computed tomography. **A** Three-dimensional view showing no graft occlusion. **B** Axial view demonstrating the omental flap between the graft and the duodenum(yellow arrow)

The patient was extubated the day after surgery. The patient’s subsequent course was excellent. There were no obvious signs of infection. Antimicrobial therapy was continued, and the patient was switched from intravenous to oral therapy. The patient was discharged home on the 30th postoperative day. Postoperative CT showed no graft occlusion and the omental flap between the graft and the duodenum (Fig. 4).

## Discussion and conclusion

Aortoenteric fistula can be further divided into primary and secondary, with primary being caused by aneurysm, infection, or trauma; secondary is defined as developing after prosthetic reconstruction, with a reported incidence of 0.36–1.6% [1, 2]. Secondary aortoenteric fistula (sAEF) is a highly lethal disease with a mortality rate of 40–70% [1], and 80% of these fistulas form in the duodenum [3].

The characteristic symptom of an aortoenteric fistula is a massive hemorrhage called a herald bleed. It precedes a fatal hemorrhage and occurs in approximately 2/3 of cases [1, 4]. In this case, the patient both had a massive hematemesis and hematochezia.

It is difficult to diagnose sAEF by upper gastrointestinal endoscopy alone, because most fistulation sites are in the horizontal part of the duodenum, and persistent active bleeding cannot be confirmed during a herald bleed. Conversely, there are many reports [5] indicating that contrast-enhanced CT is useful for diagnosis, and findings such as a soft tissue shadow, air density around a contrasted artificial blood vessel, signs of liquid effusion, and false lump formation are highly suspicious for sAEF.

It is considered important to perform surgery immediately if sAEF is suspected; however, there is no consensus regarding the optimal operative method. In principle, the operative method should be divided into aortic and duodenal. Aortic revascularization can be performed by anatomic revascularization with artificial blood vessel replacement, non-anatomic revascularization, or bridge therapy with open surgery after endovascular aortic repair (EVAR). Many recent reports suggest that anatomic revascularization is preferable [6, 7, 8]. In this case, thoracoabdominal aortic replacement was required and EVAR was not considered due to the complexity of the procedure. For the duodenum, there are several techniques, including simple suture closure of the fistula, fistulectomy plus suture closure, and resection of the horizontal part of the duodenum plus reconstruction of the gastrointestinal tract [9]. The choice of technique should be based on an overall assessment of fistula size and the degree of intra-abdominal contamination. Omental flap transposition has also been reported to be useful [9].

This one-stage, multifaceted surgical approach combined thoracoretroperitoneal incision and midline abdominal incision is safely and useful strategy. The advantage is that aortic surgery can be performed without any manipulation of the gastrointestinal tract. The risk of major bleeding can be reduced by securing the descending aorta early. In addition, since the gastrointestinal tract is not operated on concurrently, intestinal fluid does not extend into the artificial blood vessels, preventing further infection. This strategy can be used even in patients who will undergo only abdominal aortic replacement surgery without cardiopulmonary bypass.

Another advantage is that cardiovascular and gastrointestinal surgery teams do not need to operate simultaneously, allowing for a smooth handover between teams. Gastrointestinal surgery can also be performed safely since aortic replacement has already been completed　and midline abdominal incision allows gastrointestinal surgeons to perform a variety of surgical procedures.

## Conclusion

A one-stage, multifaceted surgical approach of combined thoracoretroperitoneal incision and midline abdominal incision improves aortic and gastrointestinal surgery safety for sAEF after prosthetic reconstruction of the abdominal aortic aneurysm. This method can be considered as one of the treatment options for sAEF.

## Data Availability

Data sharing is not applicable to this article as no new data were created or analyzed in this study.

## Abbreviations

sAEF: Secondary aortoenteric fistula
CT: Contrast-enhanced computed tomography
V-A ECMO: Veno-arterial extracorporeal membrane oxygenation
EVAR: Endovascular aortic repair
